# Noncommunicable Diseases and Injuries: Action Needed in South Asia Too

**DOI:** 10.1371/journal.pmed.0040038

**Published:** 2007-01-30

**Authors:** Ali Khan Khuwaja, Riaz Qureshi, Zafar Fatmi

We read with great interest the essay by Perel et al. [1] on noncommunicable diseases (NCDs) and injuries in Latin America and the Caribbean (LAC) countries. The authors are to be congratulated for their excellent description of the epidemic of NCDs and injuries in the LAC region. We wish to comment on this growing epidemic of NCDs with reference to South Asian (SA) countries, where the situation is comparable to the LAC region.

South Asia, which has one quarter of the world's population, is experiencing a rapid epidemiological transition similar to the LAC countries. The rising epidemic of NCDs in the SA region is fuelled by demographic ageing and globalization resulting in changing lifestyle, eating habits, and working patterns with less physical activity.

In 2000, 44% of the burden of disease in this region measured in disability-adjusted life years (DALYs) lost was attributed to NCDs [2], and these figures are expected to rise. Yet this growing epidemic is a neglected health issue in these countries to a greater extent. Cardiovascular diseases are the major contributors to premature mortality and morbidity in the SA region. The prevalence of diabetes has risen more rapidly in South Asia than in any region of the world. By the year 2030, India will have the highest number of persons with diabetes (79.4 million) [3]; similar trends are also projected for other SA countries. Overall, prevalence of hypertension among Pakistani adults (greater than or equal to 15 years) is about 19% [4], and this is likely to be the pattern in other SA countries. In South Asia, one third of the adult population is classified as obese and the trend is also increasing in SA children [5,6]. Large numbers of South Asians use tobacco in various forms: it is estimated that up to 65% of all men use tobacco in some form [7]. Tobacco use is responsible for approximately half of the tumors in males [8]. South Asians have one of the highest rates of oral cancers reported worldwide, and the rates are still increasing [7,8]. Due to the lack of reliable data and under-reporting of injuries, it is difficult to estimate their prevalence and future projections; nevertheless, the burden is substantially high enough to be one of the major health concerns in South Asia. In Sri Lanka alone, a smaller SA country, road traffic injuries result in 2,000 deaths and 14,000 injuries each year [9].

NCDs are expensive diseases to manage, and SA countries, which already have poor health and economic indicators, cannot afford this emerging costly epidemic. South Asians have a tendency to develop cardiovascular diseases at relatively earlier ages compared to other parts of the world, resulting in the highest potential of loss of productive life years. For a low-income Indian family with an adult with diabetes, as much as 25% of family income may be devoted to diabetes care [10].

Like the LAC region [1], SA countries have social and cultural disparities and inequalities. People of higher socioeconomic status and men who are the major economic contributors of their families are usually able to access the best available health-care facilities. As in LAC countries, South Asians of lower socioeconomic levels have the highest prevalence of mental health problems. The SA countries are well-equipped with highly qualified human resources and have common culture and languages, which can enhance more meaningful research, but are often unable to produce significant levels of quality research due to lack of funding and financial resources. With some exceptions, much of the research on NCDs has been descriptive or observation and on a small scale. Hence, the generalizability of existing research for the whole region is questionable and translating this research into practice is also difficult.

Keeping in mind the frightening scenario of NCDs in SA countries, the best option to tackle the epidemic is to take earlier action through comprehensive, multifaceted, and multicultural preventive and interventional strategies. There is also a need for more population-based local research on NCDs, with more collaboration and networking. These all require innovation, funding, political will, and health partnership between individuals, communities, clinicians, public health practitioners, nongovernmental agencies, policy makers and governments of the SA region.
